# The Influence of the Process Parameters on the Mechanical Properties of PLA Specimens Produced by Fused Filament Fabrication—A Review

**DOI:** 10.3390/polym14050886

**Published:** 2022-02-23

**Authors:** Vasile Cojocaru, Doina Frunzaverde, Calin-Octavian Miclosina, Gabriela Marginean

**Affiliations:** 1 Department of Engineering Science, Babeș-Bolyai University, P-ța Traian Vuia, Nr. 1-4, 320085 Resita, Romania; vasile.cojocaru@ubbcluj.ro (V.C.); calin.miclosina@ubbcluj.ro (C.-O.M.); 2 Department of Materials Science and Testing, Westphalian University of Applied Sciences Gelsenkirchen Bocholt Recklinghausen, Neidenburgerstr. 43, 45897 Gelsenkirchen, Germany; gabriela.marginean@w-hs.de

**Keywords:** polylactic acid (PLA), fused filament fabrication (FFF), mechanical properties, process parameters

## Abstract

Polylactic acid (PLA) is produced from renewable materials, has a low melting temperature and has a low carbon footprint. These advantages have led to the extensive use of polylactic acid in additive manufacturing, particularly by fused filament fabrication (FFF). PLA parts that are 3D printed for industrial applications require stable mechanical properties and predictability regarding their dependence on the process parameters. Therefore, the development of the FFF process has been continuously accompanied by the development of software packages that generate CNC codes for the printers. A large number of user-controllable process parameters have been introduced in these software packages. In this respect, a lot of articles in the specialized literature address the issue of the influence of the process parameters on the mechanical properties of 3D-printed specimens. A systematic review of the research targeting the influence of process parameters on the mechanical properties of PLA specimens additively manufactured by fused filament fabrication was carried out by the authors of this paper. Six process parameters (layer thickness, printing speed, printing temperature, build plate temperature, build orientation and raster angle) were followed. The mechanical behavior was evaluated by tensile, compressive and bending properties.

## 1. Introduction

Additive manufacturing (AM) technologies are increasingly used for component fabrication and tend to become an essential topic of the Industry 4.0 concept [1]. These technologies shorten the manufacturing time, thereby allowing the rapid transition from 3D models to real parts. Using additive manufacturing, both the external and the internal geometry of components can be optimized. The optimization of the internal geometry of parts allows for an efficient material distribution, correlated to the stress state.

The ISO/ASTM 52900:2015 standard [2] defines the following categories of processes used for additive manufacturing of polymers: material extrusion, material jetting, powder bed fusion, binder jetting, vat photo-polymerization and sheet lamination.

Fused filament fabrication (FFF) is a material extrusion process in which the part is built up by successive layers, each of them being made line by line. The material, in the form of a continuous filament, is melted and deposited by a printing head with a nozzle. Fused filament fabrication (also known as fused deposition modeling) is currently one of the most widely used additive technologies [1]. A great variety of equipment has been developed for the FFF technology, ranging from industrial and laboratory use to office and hobby applications.

The advantages of fused filament fabrication are based on the simplicity of the process and on the low cost of materials, equipment and consumables [3]. For the widespread use of fused filament fabrication for industrial manufacturing, it is necessary to obtain printed products with predictable properties. The following categories of AM product requirements are defined by ISO 17296-3:2014 [4]:

• Surface requirements: surface texture, appearance, color;
• Geometric requirements: linear and angular dimensions, dimensional tolerances, geometrical tolerances (deviations in shape and relative position);
• Mechanical requirements: hardness, tensile strength, impact strength, compressive strength, flexural strength, fatigue strength, creep, ageing, frictional coefficient, shear resistance and crack extension;
• Build material requirements: density, physical properties and chemical properties.

The mechanical properties of components obtained by fused filament fabrication are influenced not only by the material properties, but also by the characteristics of the 3D printer, the process parameters and the post-process treatments [5,6,7,8,9].

The 3D model conversion for the printing process is achieved by using a slicer software (a G-code generation software, specific to the printing process). This software allows for setting the values for a large number of process parameters, the most frequently analyzed being the following [6]:

• Slicing parameters: layer thickness, printing speed/flow rate, nozzle diameter, raster parameters, number of wall lines, wall thickness, top layer thickness, bottom layer thickness;
• Temperature parameters: printing head temperature, build plate temperature, build volume temperature (printer with/without closed space), cooling;
• Infill parameters: infill density and infill pattern;
• Build orientation parameters and the use of support material.

Polylactic acid (PLA) is a thermoplastic polyester that can be obtained from renewable resources at a low production cost. PLA has a low melting point, making it easy to use in most FFF equipment. The extrusion temperature of PLA is lower than that of other common polymeric materials (ABS—acrylonitrile butadiene styrene, PEEK—polyether ether ketone, PETG—polyethylene terephthalateglycol), and its tensile strength and elastic modulus may be superior to ABS and PET-G [10,11,12,13]. Furthermore, PLA is biodegradable, has a low carbon footprint and low smoke emissions during extrusion [13] and can be successfully used in medical applications, because it is not metabolically harmful [14].

The influence of the process parameters on the mechanical properties of PLA specimens obtained by fused filament fabrication has been intensively studied in recent years. In the research carried out so far, one to five process parameters have been varied. Statistical methods, such as design of experiments (DOE), the Taguchi method, and analysis of variance (ANOVA) were used to determine the influence of the different parameters on the mechanical characteristics [15,16].

In order to understand the effect of each of these numerous parameters, as well as the correlation between them, a systematic analysis of the published research is necessary. Therefore, the goal of this paper is to present an up-to-date review of the literature targeting the influence of the process parameters on the mechanical properties of PLA specimens, made by fused filament fabrication. The analysis focused on the variation of the following parameters: layer thickness, printing speed, printing head temperature, build plate temperature, build orientation and raster angle. For the characterization of the static mechanical behavior, the results of tensile, bending and compression tests were followed. The literature review was performed following the Preferred Reporting Items for Systematic Reviews and Meta-Analyses (PRISMA) guidelines.

The search terms used in bibliographic databases to select the analyzed papers were as follows: (PLA OR *poly$lactic*) AND (FDM OR FFF OR *fused*) AND (mechanical OR tensile OR bending OR strength). Papers dealing with the dynamic behavior of PLA, with PLA-based composites or only with the variation of mechanical properties as a function of the infill pattern and the infill density were not included in the present review. As the variation of mechanical properties according to the type of infill has been intensively studied, an analysis of the influence of the infill pattern and the infill density on the mechanical behavior of printed parts will be presented by the authors in a separate paper.

Table 1 shows the notations and abbreviations used in this paper to define the process parameters and mechanical characteristics.

## 2. From Pre-Process Conditions to Mechanical Characterization of FFF PLA

The systematic analysis of trends in the variation of the mechanical properties of PLA components as a function of one or more process parameters must take into consideration the values used for all factors that may influence these characteristics. Therefore, the operational conditions for all the steps involved, from filament production to mechanical testing, should be known.

In this connection, at least the following defining phases of the production and testing processes should be taken into account: (a) the manufacturing and storage conditions of the filament; (b) the design of the product and the selection of the infill parameters; (c) the selection of the process parameters; (d) type of the 3D printing equipment; (e) post-process treatments, storage conditions and ageing; and (f) the mechanical tests conditions.

### 2.1. The Manufacturing and Storage Conditions of the Filament

In fused filament fabrication processes, filaments with a circular cross-section and a diameter of 1.75 mm or 2.85 mm are used. Up to 17% variation of the ultimate tensile strength of specimens was pointed out in [18], using PLA filaments from different manufacturers. Significant differences (approximately 18% of the ultimate flexural strength) were also obtained by the bending test [19]. These differences may occur due to the manufacturing conditions or to the filament storage conditions. For example, the humidity of the filament storage enclosure can cause changes in the printing behavior and thereby noticeable variations of the mechanical properties. The color of the filament can also influence the mechanical characteristics of PLA specimens [20,21].

### 2.2. The Design of the Model and the Selection of the Infill Parameters

In the design process, the shape and dimensions of the part are determined. For 3D-printing fabrication, the output of the design must be a 3D model of the part, exported as a stl file. The quality of the stl file can influence the dimensional accuracy of the part. As the surfaces of CAD models are converted into meshes of triangles in stl files, an increase in the number of triangles leads to better quality, but also to an increase in stl file size.

The designer has also to define the infill settings that will be used. The selection of the infill pattern and density must be correlated with the stress and strain states for the future product. Both parameters are major factors of influence on the mechanical properties of 3D FFF-printed components [22,23,24,25]. The infill characteristics are to be set into the slicer software (addressed in the next step), but it is important to highlight that the infill selection is part of the design process of the component.

### 2.3. The Selection of the Process Parameters

This step involves the positioning of the part onto the printer’s build space, the choice of the values for the slicing parameters, and the setting of the temperature conditions. The G-code generated in the slicer software controls the printing process.

The selection of the process parameters must be correlated with the anisotropic behavior of PLA components manufactured by fused filament fabrication. Build orientation and raster settings have a major influence on the anisotropy of printed parts [26,27,28,29].

The designer must also consider at this stage the possibility of making the part with or without the use of support material. The use of support material can influence the surface quality and mechanical behavior of the products.

The high influence of the number of specimens printed simultaneously on the flexural strength of rectangular hollow cross-section specimens is highlighted in [30].

### 2.4. The Type of the 3D-Printing Equipment

The printing equipment can influence the dimensional accuracy and mechanical behavior of fused filament fabricated parts. Vettori and co-authors [8] present a round-robin study performed on PLA printed on different FFF equipment, using the same process parameters. The results show the important differences of the ultimate tensile strength values (max = 54.2 MPa, min = 13.2 MPa) depending on the printer used.

Temperature variations may occur in printing on open workspace equipment. Different mechanical properties can be achieved in these situations for identical components placed in different areas of the workspace. The use of closed space equipment with heat flow control can lead to optimized temperature distribution [31].

### 2.5. The Post-Process Treatments, Storage and Ageing

The mechanical behavior of FFF 3D-printed components can be influenced by post-printing thermal or thermo-chemical treatments, as well as by material ageing [32]. In [33] is highlighted the improvement of thermomechanical properties of PLA specimens subjected to post-print annealing. However, in [34] it is shown that PLA specimens obtained by FFF and annealed at 60–120 °C for 30–60 min showed a decrease in the modulus of elasticity and the ultimate tensile strength.

The properties of components made of PLA can be modified by the storage environmental conditions and the storage duration. Moreover, the dimensional accuracy of printed PLA components can be influenced by material volume changes and residual stress occurrence caused by the PLA crystallinity [35].

At low humidity, PLA has higher mechanical strength but lower toughness [36]. In [37] it is shown that reducing moisture content from 10% to 1% results in a decrease in the tensile strength with 24.4%.

In [38] are presented the variations of yield strength and modulus of elasticity as functions of the ageing time for printed PLA. An improvement of the mechanical characteristics is observed with an increase in the ageing duration. Contrariwise, ageing for 240 h in a salt-fog environment causes a decrease of about 20% of the tensile strength [39].

### 2.6. The Mechanical Tests Conditions

The most commonly used testing methods for the characterization of the mechanical behavior of fused filament fabricated PLA parts are tensile tests and three-point bending tests [40]. At the moment, there are no specific ISO or ASTM standards defining the shape of specimens manufactured by FFF additive manufacturing. Thus, for the tensile tests “dog-bone” specimens defined by the general standards for plastics are used: ASTM D638-14 [41] and ISO 527-2:2012 [42]. The specimens used for the bending tests are defined by: ASTM D790-10 [43] and ISO 178:2019 [44].

The use of specimens with different shapes and dimensions may result in different mechanical characteristics. In [28], the authors estimate that the UTS values obtained by tests made on ASTM D638—Type IV specimens may be overestimated compared to the values resulting from tests performed on ASTM D638—Type I specimens.

One of the main problems highlighted in several papers refers to the occurrence of the breakage outside the gauge length of the tensile specimens. This improper failure may be related to the geometry of the dog-bone specimen, which leads to stress concentrations in the radius area [1]. Sierra et al. [45] studied the tensile behavior of ASTM D638 specimens with modified radius and conclude that the radius influences the mechanical strength obtained in tests. In [1] it is shown that an alternative to specimens with radius is the use of prismatic specimens defined by ISO 527-5 [46] and ASTM D3039 [47].

Valean et al. show that increasing the thickness of the specimen decreases the values of the mechanical characteristics UTS and E determined by tensile tests [48].

The variation of the mechanical properties of PLA specimens depending on the tensile test speed and the tensile test strain rate was analyzed in [49,50]. Vidakis and coauthors conclude that the tensile strength of PLA is strongly influenced by the strain rate and tensile test speed. The increase in the test speed from 10 mm/min to 100 mm/min leads to an increase in the tensile strength values by approximately 11% [50].

## 3. Layer Thickness

**Figure 1:**
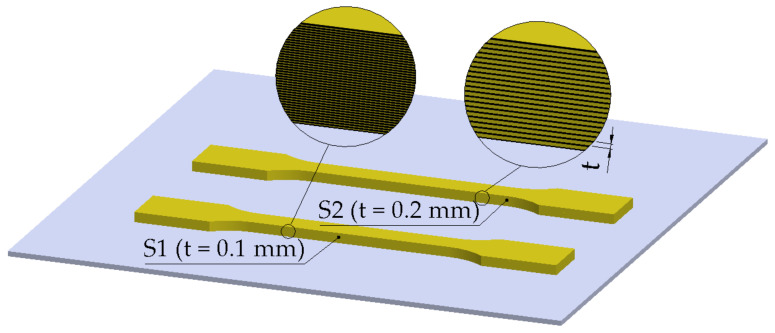
Layer thickness (t) for ISO 527-2 Type 1A tensile test specimens (S1, S2).

The layer thickness (or layer height) is the height of each deposited layer (Figure 1). For the top and the bottom layer, respectively, a distinct thickness can be set. It should be noted that the layer thickness is correlated with the diameter of the nozzle and the width of the raster.

In the research analyzed in this paper, the layer thickness was varied in the range of 0.06–0.6 mm, with the most commonly analyzed values situated between 0.10 mm and 0.30 mm. Selecting higher values for layer thickness leads to shorter production times, but also to lower part resolution. On the other hand, working with lower layer thicknesses determines longer durations of the printing processes and higher part resolution. The total number of layers is the ratio of the part height on the z-axis to the layer thickness (reference system for upward building, according to ISO/ASTM 52921:2013). A 50% decrease in the layer thickness results in a doubling of the printing time. Increasing the number of layers emphasizes the re-heat effect for deposited layers, leading to improved diffusion and adhesion between layers. 

It should be noted that the variation of the mechanical properties with the layer thickness is influenced also by other parameters (Table 2). For example, in [51] it is shown that the dependence of the tensile strength on the layer thickness is affected by the raster type. On the other hand, the influence of the nozzle diameter of the printing head is greater than the influence of the layer thickness when a high yield strength is desired for a product [52]. Triyono et al. [53] indicate that the increase in the nozzle diameter leads to an increase in the density and the tensile strength of 3D-printed products. The authors consider that these two interconnected effects can be attributed to better interfacial bonding between the in-plane raster lines. At the same ratio between layer thickness and nozzle diameter, the adhesion between adjacent lines improves with the increases in the nozzle diameter. For big nozzle diameters, the raster lines were discovered to be even slightly overlapped.

## 4. Printing Speed

The printing speed (mm/s) is the speed of the printing head in the XY plane during the deposition of the layers. This parameter is correlated with the flow rate (mm^3^/s).

In the research analyzed in this paper (Table 3) the printing speed was varied in the range of 20 mm/s–170 mm/s. The increase in the printing speed leads to a decrease in the part manufacturing duration but worsens the dimensional accuracy. High printing speeds reduce the degree of solidification of the bottom layers at the deposition of new layers. This can cause sliding processes between the successive deposited layers (mainly at the edges of the part) and thereby significant dimensional deviations.

## 5. Printing Head Temperature and Build Plate Temperature

The printing head temperature is one of the most studied process parameters. As revealed by Table 4, the researchers selected printing head temperatures ranging from 175 °C to 275 °C for manufacturing of the PLA samples, but the most commonly analyzed temperatures were situated between 190–220 °C. These values correlate with the melting point of PLA (160 °C up to 180 °C). The tendency to use lower temperatures is associated with the susceptibility of the PLA to thermal degradation at high temperatures and with economic issues (reduced energy consumption). At the same time, at low printing temperatures (below 180 °C, according to [34]), melting may not be complete and interlayer diffusion may not occur. Low diffusion between layers can cause delamination (peeling of layers). In [89] it is shown that at low printing temperatures the air gaps between raster lines are larger, which leads to reduced tensile strength.

Higher printing head temperatures can provide better interlayer diffusion and higher mechanical properties, but also a slip of the deposited material, affecting the dimensional accuracy of the components. In [34] it is shown that the use of printing temperatures above 240 °C causes an unsteady flow of material from the printing head nozzle.

The build plate temperature is generally set in the range of 50–60 °C. In open-space 3D printers, the uniformity of the build plate temperature is difficult to achieve because of the heat flows. In general, in the central areas of the build plate the temperature is higher compared to the peripheral areas. This disadvantage is mitigated for the printers by closed work space and controlled heat flow. In [60] it is shown that the influence of the heat flux on the ultimate tensile strength is low when the specimens are printed horizontally and high when the specimens are printed vertically.

Considering both temperature-related parameters—the printing head temperature and the build plate temperature, respectively—it is shown that the influence of the printing head temperature on the mechanical properties is lower compared to the influence of the build plate temperature [33].

The importance of temperature profile monitoring during the FFF-printing process by using specific devices (infrared camera, thermocouples) and the development of numerical heat transfer models is highlighted in [96].

## 6. Build Orientation of the Specimens

**Figure 2:**
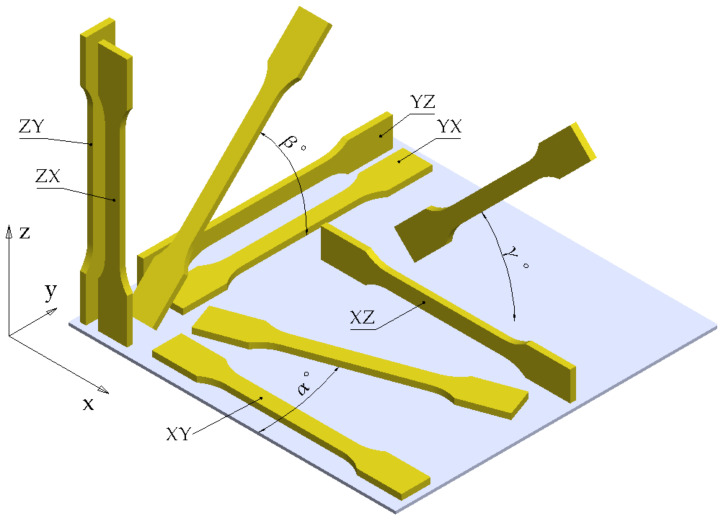
Notation of different build orientations.

The placement of the 3D model onto the building space of the printer is one of the main factors that determine the anisotropic behavior of PLA FFF-printed parts. In this regard, high differences were found between the mechanical behavior along the x and y axes (axes situated in the plane of the build plate—Figure 2) and the mechanical behavior along the vertical z-axis. Variations of mechanical properties for the parts rotated with various angles to the reference system must also be included in the analysis.

In the ISO/ASTM 52921:2013 standard [17] the notation of the orthogonal orientation (non-rotated) of a prismatic part relative to the printer reference system is done by combinations of three letters: the first letter of the notation represents the axis parallel to the longest characteristic dimension of the part, the second letter represents the axis parallel to the second-longest characteristic dimension of the part and the third letter represents the axis parallel to the third characteristic dimension. If the part has a symmetry plane (as in the case of dog-bone tensile specimens), a simplified notation consisting of the first two letters may be used.

Figure 2 shows the notation of the positioning of an ISO 527–2:2012 Type 1A tensile specimen. The first characteristic dimension is the length of the specimen and the second characteristic dimension is the width of the specimen. The necessity of using standardized notations for build orientation results from the analysis of the articles published so far (Table 5). In several articles, XY and YX build orientations are referred to as “flat build orientations”, XZ and YZ build orientations are referred to as “on-edge build orientations” and ZX and ZY build orientations are referred to as “upright build orientations”. The use of the term “flat build orientation”, without graphic detail, does not clearly indicate whether XY or YX build orientation is used. The ambiguity is amplified in the cases where rotated specimens relative to the orthogonal orientation are used. In this paper we propose the use of angles α, β and γ for describing rotations in the xy, yz and zx planes. To define the angle of rotation relative to an orthogonal orientation, indices will be used (angle α_XZ_ defines a specimen rotated by α° in the xy plane relative to the base orientation XZ, angle α_XZ_ = 0° represents the XZ orthogonal orientation and α_XZ_ = 90° represents the YZ orientation). For a comparative analysis, the notations from Figure 2 were used for the papers listed in Table 5. For some papers, where it was not possible to unambiguously identify the build orientation, the notations given by the authors were maintained.

Analyzing the data presented in Table 5, it can be concluded that the ZX- and ZY-type build orientations lead to much lower mechanical characteristics compared to the XY, YX, XZ and YZ layouts. This mechanical behavior is generated by the inter-layer breakage that occurs in ZX and ZY specimens.

At tilted specimens relative to the build plate (0° < β_YZ_ < 90°; 0° < β_YX_ < 90°; 0° < γ_XZ_ < 90°; 0° < γ_XY_ < 90°), the mechanical characteristics decrease with increases in the tilt angle.

A comparative analysis of the XY and the YX build orientations should be correlated with the raster angle (similar for specimens with 0° < α_XY_ < 90°).

The anisotropic character of components made by FFF printing was also evidenced by some authors through mechanical tests performed on specimens obtained by conventional machining (cutting) from 3D-printed prismatic blocks [105].

## 7. Raster Angle

**Figure 3:**
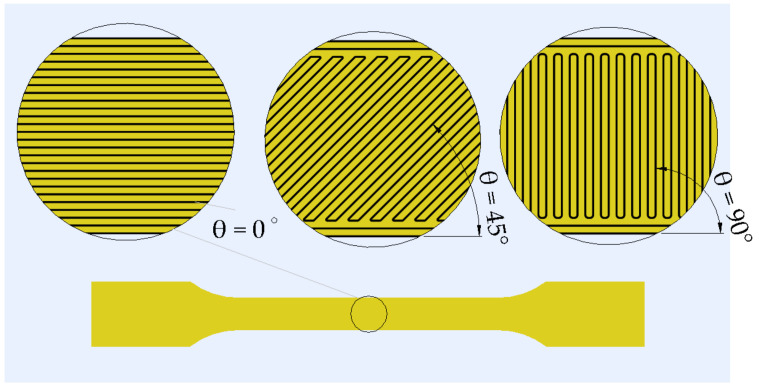
Raster angle (θ).

The raster of the 3D-printed parts represents the arrangement of the successive lines of a layer (Figure 3). The mechanical behavior is influenced by several raster parameters: the raster angle, how the raster angle alternates between two successive layers, the width of a raster line, the distance between two successive raster lines, the number of wall lines and the distance between the raster and the wall lines [106].

The raster angle influences the anisotropic mechanical behavior and the breakage of 3D FFF-printed components. Two main types of layouts are distinguished: unidirectional raster (the same raster angle is maintained for all successive layers) and alternating raster (the raster angle varies between successive layers, usually by 90°). Even in the case of the raster angle, a standardization of notations is needed with a clear identification of the alternating raster. Therefore, notations in the form of θ_1_°/ θ_2_° could be used, where θ_1_° and θ_2_° represent the raster angles for two successive layers.

In the previous research were analyzed specimens with unidirectional raster and alternating raster (Table 6—the first 11 lines of the table show unidirectional raster, the next 14 lines of the table show alternating raster). The highest mechanical properties were obtained for the specimens with alternating raster. The analysis of the mechanical behavior as a function of the angle of the raster should be carried out correlated with the specimen build orientation.

**Figure 4:**
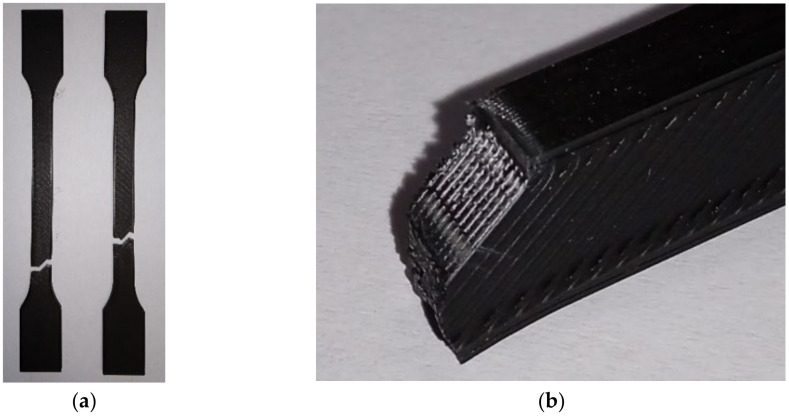
Breaking surface aligned with the raster angle; (**a**) ISO 527-2:2012 1A specimens; (**b**) detail.

The failure of tensile specimens can be influenced by raster and build orientations. Three failure modes can be defined:

• Inter-layer failure, when the failure occurs at the interface between two adjacent layers [77] (ex. the breaking of tensile specimens with ZY or ZX orientations);
• Inter-line failure (the breaking surface aligned with the raster angle—Figure 4);
• In-layer failure or in-line failure (the breaking surface is not aligned with the raster angle or the interface between two adjacent layers).

Inter-line failure can be associated with reduced diffusion between the raster lines.

The occurrence of inter-line failure at the XY specimens with a unidirectional raster angle of θ = 90° is the cause for the lower tensile strength of these specimens relative to the tensile strength of specimens with θ = 0° or θ = 45°.

The effect of stress concentration in the radius area of the tensile specimens may be amplified by the raster layout, mainly in the case of the unidirectional raster [1].

The optimization of the fused filament fabrication technology certainly has to start with the prioritization of the process parameters according to their impact on the mechanical properties of the printed part. In [22] a hierarchy of the influence of six process parameters on several mechanical properties is presented. For specimens printed in the XZ orientation, the order of the influence of the process parameters on the ultimate tensile strength is considered to be: infill density, layer thickness, presence of a contour wall, head temperature, infill orientation and printing speed, while in case of the XY orientation the order of the importance of these parameters is different: layer thickness, infill orientation, infill density, head temperature, printing speed and presence of a contour wall. This order changes when other mechanical parameters (Young’s modulus, yield strength, etc.) are monitored.

## 8. Discussions and Conclusions

Fused filament fabrication is a widespread technology, used in various applications, ranging from industrial manufacturing and research activities to home use. Polylactic acid is a biodegradable, low-carbon-footprint material that can be used for the fabrication of industrial products if predictable and repeatable mechanical properties are achieved in the production process.

The mechanical behavior of components made of PLA by FFF is influenced by several factors along the production chain: filament manufacturing, geometrical design, process parameters, 3D-printing equipment, ageing and post-process treatments and mechanical testing procedure.

From the process parameters most investigated in the literature, in this paper the following have been analyzed: layer thickness, printing speed, printing head temperature, build plate temperature, build orientation and raster angle.

The necessity for standardization and uniformity in the definition of process parameters is highlighted. Comparative analysis of previous research is hampered by ambiguous or incomplete definitions of certain process parameters. Furthermore, the simultaneous variation of several process parameters during the experimental investigations conduces to difficulties in pointing out the influence of each parameter considered individually.

Finally, the critical need to define suitable specimens for the mechanical testing of FFF products is revealed by the large number of tensile specimens with breakage occurring outside the gauge length. Without specific regulations, in order to reduce the errors caused by failure outside the calibrated area, testing of a higher number of specimens may be considered.

The results presented in the literature indicate that at lower layer thicknesses better interlayer diffusion is achieved, the air voids are smaller, the surface quality is better, and the mechanical properties are higher.

High printing speeds can lead to an inadequate surface quality because of the incomplete solidification of the underlying layers when the top layers are deposited.

Low printing head temperatures can cause incomplete melting, while high printing temperatures can cause unstable material flow from the printing head. Controlling heat flows by using enclosed workspace equipment can reduce the temperature gradients on the build plate. 

Upright printed specimens (ZY and ZX build orientations) have considerably lower mechanical strength compared to horizontally printed specimens (XY, XZ, YX and YZ). The mechanical properties decrease with increases in the specimen positioning angle relative to the build plate.

The use of the alternating raster leads to superior mechanical properties compared to the unidirectional raster. The anisotropic behavior of PLA components made by fused filament fabrication is highly correlated with the raster parameters, build orientation and the type of failure: inter-layer failure, inter-line failure and in-layer/in-line failure.

## Figures and Tables

**Table 1:** Notations and abbreviations.

Process Parameter	Notation	Units
Layer thickness (layer height)	t	(mm)
Printing speed	s_p_	(mm/s)
Printing head (nozzle) temperature	T_H_	(°C)
Build plate temperature	T_B_	(°C)
Nozzle diameter	d_n_	(mm)
Filament diameter	d_f_	(mm)
Build orientation (acc. to ISO/ASTM 52921:2013 [17]) first letter—axis parallel to the longest dimension of part; second letter—axis parallel to second longest dimension of part	XY, XZ, YX, YZ, ZX, ZY	(-)
Build orientation angle in the xy plane (around the z-axis) Indexes represent the reference build orientation from which angle is measured (α_ZX_ = 0° correspond to ZX build orientation);	α_YX,_ α_XY,_ α_XZ_	(°)
Build orientation angle in the yz plane (around the x-axis)	β_XY,_ β_YX,_ β_XZ_	(°)
Build orientation angle in the xz plane (around the y-axis)	γ_ZX,_ γ_XY,_ γ_XZ_	(°)
Raster angle	θ	(°)
Number of wall lines	W_L_	(-)
Tensile/bending test speed	s_t_	(mm/min)
Ultimate tensile strength	UTS	(MPa)
Ultimate flexural strength	UFS	(MPa)
Modulus of elasticity (Young’s modulus)	E	(MPa)

**Table 2:** The influence of the layer thickness on the mechanical properties of FFF-printed PLA.

Ref.	FFF Process Parameters							Mechanical Test Settings	Results and Conclusions
	t (mm)	s_p_ (mm/s)	T_H_ (°C)	T_B_ (°C)	B.O. (-)	θ (°)	Other Parameters		
t—layer thickness (layer height); s_p_—printing speed; T_H_—printing head (nozzle) temperature; T_B_—build plate temperature; B.O.—build orientation; θ—raster angle; d_f_—filament diameter; d_n_—nozzle diameter; W_L_—number of wall lines.									
[19]	0.06–0.60	25	-	-	Vertical	-	d_f_ = 2.85 mm; d_n_ = 0.4–0.8 mm	Bending, rectangular hollow cross-section; s_t_ = 10 mm/min	UFS increases with the increase in the d_n_/t ratio. UFS for t = 0.06, d_n_ = 0.40 about 3.9× higher than UFS for t = 0.4, d_n_ = 0.40.
[23]	0.10–0.20	20–40	210	-	XY	45°/−45°	d_f_ = 1.75 mm; d_n_ = 0.4 mm; 20–80% infill	Tensile—ASTM D638	Low increase in UTS with the decrease in layer thickness.
[24]	0.10–0.30	30	195	110	Horizontal	40°–80°	d_f_ = 1.75 mm; d_n_ = 0.3 mm; 20–80% infill	Tensile—ASTM D638	The variation of UTS vs. layer thickness is influenced by the raster angle.
[26]	0.06–0.24	20–80	210	-	YX; YZ; ZY	0°	d_f_ = 1.75 mm; d_n_ = 0.4 mm; 100% infill	Tensile—ASTM D638; Bending—ASTM D790	Highest UTS (89.1 MPa) for t = 0.06, s_p_ = 50 mm/s, YX specimens. Highest UFS (65 MPa) for t = 0.06, s_p_ = 80 mm/s, YZ specimens.
[28]	0.06–0.50	30–200	175–230	-	XY; ZX	-	d_f_ = 1.75 mm; d_n_ = 0.5 mm; 100% infill	Tensile—ASTM D638, Type I vs. Type IV	UTS decreases with the increase in the layer thickness.
[32]	0.10–0.30	60	215	60	Horizontal	45°/−45°; 0°/90°; 0°/−30°/ 30°/−60°/ 60°/90°/	d_f_ = 1.75 mm; 100% infill; ageing; heat treatment	Tensile—ASTM D638	Higher UTS for specimens with t = 0.1 mm. The decrease in UTS for t = 0.3 mm vs. t = 0.1 mm is higher for aged specimens, with and without heat treatment.
[51]	0.06–0.35	60	190–220	60	XY	0°; 90°; 45°/−45°	d_f_ = 1.75 mm; d_n_ = 0.4 mm; 100% infill; W_L_ = 2	Tensile—ASTM D638, Type I specimens; s_t_ = 5 mm/min	The variation of UTS with layer thickness is influenced by θ. For θ = 0° the highest UTS is obtained for t = 0.06 mm. High variation of UTS vs. t for θ = 90°.
[54]	0.10–0.40	90	185	-	Z	-	d_f_ = 1.75 mm; 100% infill	Tensile—ASTM D638 IV; s_t_ = 5 mm/min	Highest UTS and E for t = 0.4 mm.
[55]	0.20–0.40	50	190–210	-	Horizontal	-	d_f_ = 2.85 mm; 20–100% infill; W_L_ = 2	Tensile—ASTM D638; increased specimen thickness; s_t_ = 5 mm/min	Highest UTS (61.66 MPa) and E (3815.50 MPa) for t = 0.3 mm.
[56]	0.10–0.40	50–150	190–205	-	Horizontal	-	d_f_ = 1.75 mm; d_n_ = 0.4 mm; cooling fan	Tensile; s_t_ = 5 mm/min	Highest UTS (60.26 MPa) for t = 0.10 mm; layer thickness was the dominant factor for UTS.
[57]	0.10–0.30	50	210	60	α_XY_ = 0°–60°	-	d_f_ = 1.75 mm; d_n_ = 0.4 mm; 20–80% infill; W_L_ = 2	Tensile—ASTM D638; Bending—ASTM D790; s_t_ = 1 mm/min	Highest UTS obtained for t = 0.2 mm and α_XY_ = 30° at 80% infill density; Highest UFS obtained for t = 0.3 mm and α_XY_ = 0° at 80% infill density.
[58]	0.125–0.25	-	-	-	α_XY_ = 0°; α_XY_ = 45°	-	50–90% infill	Tensile—ISO 527	Higher UTS for t = 0.25 mm.
[59]	0.10–0.35	40–80	220	25	α_XY_ = 0°–90°	-	d_f_ = 1.75 mm; 100% infill	Tensile—ASTM D638, Type V specimens	Higher E and UTS for low values of the layer thickness.
[60]	0.05–0.40	60	200	-	Horizontal; Vertical	-	d_f_ = 1.75 mm; 60% infill; variable cooling	Tensile	Highest UTS (53.62 MPa) at t = 0.2 mm, for horizontal printed specimens.
[61]	0.20–0.30	38–52	190	40	-	0°; 90°	d_n_ = 0.40 mm; 40% infill	Bending—ASTM D790; s_t_ = 12 mm/min	Higher flexural strength for t = 0.2 mm.
[62]	0.10–0.30	25–75	210	60	Vertical	-	d_n_ = 0.40 mm; four FFF printers (P1-P4)	Bending, rectangular hollow cross-section; s_t_ = 10 mm/min	P1-P2: UFS and sample mass decrease with thickness. P3-P4: maximum UFS for t = 0.15 mm and t = 0.20 mm.
[63]	0.10–0.20	60	205	60	Horizontal	0°; 18°; 45°; 72°; 90°	100 infill; W_L_ = 2–6	Tensile—ASTM D638 modified specimens	Low variation of UTS and E with t. Highest UTS (49.29 MPa) and E (3497 MPa) for t = 0.10 mm.
[64]	0.10–0.30	-	210	80	γ_XY_ = 0°–90°	30°; 45°; 60°	d_f_ = 1.75 mm; 50% infill	Tensile—ASTM D638	UTS decreases with the increase in the layer thickness.
[65]	0.10–0.30	30–90	210–230	50–80	XY	0°/90°	d_f_ = 1.75 mm; d_n_ = 0.4 mm; 100% infill; W_L_ = 2	Tensile—ISO 527–2; s_t_ = 50 mm/min	Higher UTS for t = 0.2 mm.
[66]	0.10–0.20	40–80	220	60	XY; XZ	-	d_n_ = 0.4 mm; 100% infill; W_L_ = 3	Tensile—ISO 527; s_t_ = 5 mm/min	Higher UTS (46.22 MPa) for XZ specimens with t = 0.1 mm, s_p_ = 80 mm/s.
[67]	0.10–0.20	60	200	60	Horizontal	-	d_f_ = 1.75 mm; d_n_ = 0.4 mm; 50–100% infill	Tensile—ISO 527–2	Low variation of UTS and E with the layer thickness. Higher UTS for t = 0.1 mm.
[68]	0.10–0.40	60	230	80	Horizontal	-	d_f_ = 1.75 mm; 100% infill	Tensile—ASTM D638; Bending—ASTM D790; Impact—ISO 180	UTS, UFS and Izod impact strength decrease with the increase in layer thickness for all raster patterns.
[69,70,71]	0.10–0.30	50	210	70	-	0°; 45°; 90°	d_f_ = 1.75 mm; d_n_ = 0.4 mm; 100% infill; W_L_ = 1	Tensile—ASTM D638; Bending—ASTM D790; Impact—ASTM D256	UTS and UFS decrease with the increase in the layer thickness. Izod impact strength increases with the layer thickness.
[72]	0.10–0.20	30	200	50	XY; XZ; ZX	45°/−45°	d_f_ = 1.75 mm; d_n_ = 0.4 mm; 20–50% infill	Tensile—ASTM D638; s_t_ = 5 mm/min	Approx. 10.6% higher UTS for t = 0.10 mm compared to t = 0.20 mm.
[73]	0.10–0.30	20	210	50	-	-	d_f_ = 1.75 mm	Tensile, s_t_ = 1 mm/min	Higher UTS (61.5 MPa) for t = 0.30 mm.
[74]	0.05–0.20	60	195–230	60	β_YZ_ = 0°–90°	-	d_f_ = 1.75 mm; d_n_ = 0.4 mm	Tensile—ISO 527–2; s_t_ = 2 mm/min	Low decrease in UTS with the increase in the layer thickness.
[75]	0.10–0.20	80	200	60	XY	45°	d_f_ = 1.75 mm; 25–100% infill; variable flow rate	Tensile—ASTM D638, Type IV	Highest UTS (40.07 MPa) for t = 0.15 mm.
[76,77]	0.10–0.30	60	215	-	γ_XZ_ = 0°–90°	-	d_f_ = 1.75 mm	Tensile—ISO 527–2; s_t_ = 0.1 mm/min	Highest UTS for t = 0.10 m. Low variation of UTS and E with layer thickness.
[78]	0.10–0.60	-	-	-	γ_XZ_ = 0°–90°	-	d_f_ = 1.75 mm; d_n_ = 0.4 mm	Tensile—ISO 527–2; s_t_ = 0.1 mm/min	Low variation of UTS with layer thickness.
[79]	0.10–0.30	-	220	60	γ_XZ_ = 0°–90°	-	d_f_ = 1.75 mm	Tensile—ISO 527–2	Highest UTS for t = 0.10 mm and t = 0.20 mm. Low variation of UTS vs. t.

**Table 3:** The influence of the printing speed on the mechanical properties of FFF-printed PLA.

Ref.	FFF Process Parameters							Mechanical Test Settings	Results and Conclusions
	s_p_ (mm/s)	t (mm)	T_H_ (°C)	T_B_ (°C)	B.O. (-)	θ (°)	Other Parameters		
s_p_—printing speed; t—layer thickness (layer height); T_H_—printing head (nozzle) temperature; T_B_—build plate temperature; B.O.—build orientation; θ—raster angle; d_f_—filament diameter; d_n_—nozzle diameter; W_L_—number of wall lines.									
[23]	20–40	0.10–0.20	210	-	XY	45/−45°	d_f_ = 1.75 mm; d_n_ = 0.4 mm; 20–80% infill	Tensile—ASTM D638	Low increase in UTS with the decrease in printing speed.
[26]	20–80	0.06–0.24	210	-	YX; YZ; ZY	0°	d_f_ = 1.75 mm; d_n_ = 0.4 mm; 100% infill	Tensile—ASTM D638; Bending—ASTM D790	The variation of UTS vs. s_p_ is influenced by the build orientation and the layer thickness.
[29]	20–80	0.40	215	55	Horizontal	0°; 30°; 45°; 60°; 90°	100% infill; W_L_ = 2	Tensile—ASTM D638; s_t_ = 5 mm/min	Higher E and UTS values for s_p_ = 20 mm/s.
[30]	12.5–50	0.30	190–250	60	Vertical	-	d_f_ = 2.85 mm; d_n_ = 0.6 mm; variable cooling	Bending, rectangular hollow cross-section; s_t_ = 10 mm/min	For T_H_ = 210 °C highest UFS (56.3 MPa) at s_p_ = 25 mm/s; high influence of s_p_ on the specimen mass.
[54]	70–170	0.30	185	-	Z	-	d_f_ = 1.75 mm; 100% infill	Tensile—ASTM D638 IV; s_t_ = 5 mm/min	Low variations of UTS and E with printing speed.
[56]	50–150	0.10–0.40	190–205	-	-	-	d_f_ = 1.75 mm; d_n_ = 0.4 mm	Tensile; s_t_ = 5 mm/min	Higher UTS for s_p_ = 80mm/s and s_p_ = 100 mm/s.
[59]	40–80	0.10–0.35	220	25	α_XY_ = 0°–90°	-	d_f_ = 1.75 mm; 100% infill	Tensile—ASTM D638 Type V specimens	Higher E and UTS values for low printing speed.
[61]	38–52	0.20–0.30	190	40	-	0°; 90°	d_n_ = 0.40 mm; 40% infill	Bending—ASTM D790; s_t_ = 12 mm/min	Higher flexural strength for s_p_ = 38 mm/s.
[62]	25–75	0.10–0.30	210	60	Vertical	-	d_n_ = 0.40 mm; 4 FFF printers	Bending, rectangular hollow cross-section	Higher UFS for s_p_ = 25 mm/s.
[65]	30–90	0.10–0.30	210–230	50–80	XY	0°/90°	d_f_ = 1.75 mm; d_n_ = 0.4 mm; W_L_ = 2	Tensile—ISO 527–2; s_t_ = 50 mm/min	Low decrease in UTS with the increase in the printing speed.
[73]	20–60	0.20	210	50	-	-	d_f_ = 1.75 mm	Tensile; s_t_ = 1 mm/min	Higher UTS for s_p_ = 20 mm/s.
[80]	40–50	0.20	190–230	50	XY	45°	d_f_ = 1.75 mm; d_n_ = 0.4 mm; 100% infill	Tensile—ASTM D638 Type IV specimens	Higher UTS values for s_p_ = 50 mm/s (except the T_H_ = 230 °C specimens).
[81]	50–150	-	190–210	-	Horizontal	-	20–100% infill	Tensile—ASTM D638 Type V specimens	Highest UTS (45.27 MPa) obtained for s_p_ = 100 mm/s and T_H_ = 210 °C.
[82]	60–100	0.10–0.30	-	-	Horizontal	-	60–100% infill	Tensile—ASTM D638; Bending—ASTM D790	Infill density and printing speed have the highest influence on UFS and UTS.
[83]	20–60	0.08–0.28	210–220	-	XY; XZ	0°/90°; 30°/−60°; 45°/−45°	d_n_ = 0.3–0.5 mm; 80–100 % infill; W_L_ = 2–4	Tensile—ASTM D638-I; s_t_ = 5 mm/min	Higher UTS for s_p_ = 20 mm/s. The optimum parameters for UTS: s_p_ = 20 mm/s, T_H_ = 220 °C, XZ orientation, 30°/−60° raster.
[84]	40–140	0.10	210	50	-	-	100% infill; W_L_ = 2; variable flow rate	Tensile—GB/T 11997 type-A specimens; s_t_ = 5 mm/min	Low influence of the printing speed. High influence of the flow rate.
[85]	35–45	0.20	180–220	25	XY	45°/−45°	d_f_ = 1.75 mm; d_n_ = 0.4 mm; 20% infill	Tensile—ASTM D638; Bending—ASTM D790; Compression—ASTM D3410; s_t_ = 5 mm/min	Tensile: higher UTS for s_p_ = 45 mm/s and s_p_ = 40 mm/s at T_B_ = 200–220 °C. Bending: higher UFS for s_p_ = 45 mm/s. Compression: higher strength for s_p_ = 45 mm/s;
[86]	35–65	0.10	200	60	XY	45°/−45°; 0°/90°	d_f_ = 2.85 mm; 100% infill	Tensile—ASTM D638	Decrease in UTS with the increase in the printing speed.
[87]	50–100	0.10–0.20	210	60	Vertical	-	40–80% infill	Bending, circular hollow cross-section specimens	Higher UFS for low printing speed and low layer thickness.
[88]	30–40	-	180–195	-	-	45°/−45°; 30°/−60°; 0°/90°	-	Tensile—ASTM D638; s_t_ = 5 mm/min; Bending—ASTM D790; s_t_ = 2 mm/min	The optimum parameters for tensile test: s_p_ = 40 mm/s, T_H_ = 180°, θ = 30°/−60°. The optimum parameters for bending test: s_p_ = 30 mm/s, T_H_ = 185°, θ = 30°/−60°.

**Table 4:** The influence of the head temperature and build plate temperature on the mechanical properties of FFF-printed PLA.

Ref.	FFF Process Parameters							Mechanical Test Settings	Results and Conclusions
	T_H_ (°C)	T_B_ (°C)	t (mm)	s_p_ (mm/s)	B.O. (-)	θ (°)	Other Parameters		
T_H_—printing head (nozzle) temperature; T_B_—build plate temperature; t—layer thickness (layer height); s_p_—printing speed; B.O.—build orientation; θ—raster angle; d_f_—filament diameter; d_n_—nozzle diameter; W_L_—number of wall lines.									
[30]	190–250	60	0.30	12.5–50	Vertical	-	d_f_ = 2.85 mm; d_n_ = 0.6 mm; variable cooling	Bending, rectangular hollow cross-section; s_t_ = 10 mm/min	Increase in ultimate flexural strength and specimen mass with the printing head temperature.
[33]	190–230	45–105	-	50	-	0°/90°; 15°/75°; 30°/60°; 45°/45°	d_f_ = 2.85 mm; 100% infill	Tensile—ASTM D638; st = 5 mm/min Bending—ASTM D790; Impact—ASTM D256	Mechanical parameters increase with T_B_. The influence of T_H_ is lower compared to the influence T_B_.
[34]	180–240	-	0.10	60	Horizontal	-	d_f_ = 1.75 mm; annealing	Tensile—ISO527; s_t_ = 5 mm/min	Increase in UTS and E with T_H_ for specimens without annealing.
[51]	190–220	60	0.06–0.35	60	XY	0°; 90°; 45/−45°	d_f_ = 1.75 mm; d_n_ = 0.4 mm; W_L_ = 2	Tensile—ASTM D638-I specimens; s_t_ = 5 mm/min	Highest UTS values for T_H_ = 220 °C and T_H_ = 205 °C. High variation of UTS vs. T_H_ for θ = 90°.
[54]	175–205	-	0.30	90	Z	-	d_f_ = 1.75 mm; 100% infill	Tensile—ASTM D638 Type IV; s_t_ = 5 mm/min	Highest UTS (43.79 MPa) at T_H_ = 205 °C. Approx. 35% increase in UTS for T_H_ = 205 °C, compared to T_H_ = 175 °C.
[55]	190–210	-	0.20–0.40	50	Horizontal	-	d_f_ = 2.85 mm; 20–100% infill; W_L_ = 2	Tensile—ASTM D638; increased specimen thickness; s_t_ = 5 mm/min	Highest UTS for T_H_ = 210 °C and T_H_ = 200 °C.
[56]	190–205	-	0.10–0.40	50–150	Horizontal	-	d_f_ = 1.75 mm; d_n_ = 0.4 mm; cooling fan	Tensile test; s_t_ = 5 mm/min	Higher UTS obtained for T_H_ = 210 °C and active cooling fan; higher T_H_ recommended for high layer thickness.
[65]	210–230	50–80	0.10–0.30	30–90	XY	0°/90°	d_n_ = 0.4 mm; d_f_ = 1.75 mm; 100% infill; W_L_ = 2	Tensile—ISO 527–2; s_t_ = 50 mm/min	Low increase in UTS with the increase in T_H_ and decrease in T_B_.
[73]	200–230	50–70	0.20	20	-	-	d_f_ = 1.75 mm	Tensile; s_t_ = 1 mm/min	Highest UTS (62 MPa) for T_H_ = 220 °C; Low variation of UTS vs. T_B._
[74]	195–230	60	0.05–0.20	60	β_YZ_ = 0°–90°	-	d_f_ = 1.75 mm; d_n_ = 0.4 mm	Tensile—ISO 527–2; s_t_ = 2 mm/min	Higher UTS for T_B_ = 210–215°C.
[80]	190–230	50	0.20	40–50	XY	45°	d_f_ = 1.75 mm; d_n_ = 0.4 mm; 100% infill; W_L_ = 3	Tensile—ASTM D638 Type IV specimens	Approx. 20% increase in UTS for T_H_ = 210 °C, compared to T_H_ = 190 °C.
[81]	190–210	-	-	50–150	Horizontal	-	20–100% infill	Tensile—ASTM D638 Type V specimens	Highest UTS (45.27 MPa) obtained for s_p_ = 100 mm/s and T_H_ = 210 °C.
[83]	210–220	-	0.08–0.28	20–60	XY; XZ	0°/90°; 30°/−60°; 45°/−45°	d_f_ = 1.75 mm; d_n_ = 0.3–0.5 mm; 80–100% infill; W_L_ = 2–4	Tensile—ASTM D638-I; s_t_ = 5 mm/min	Higher UTS for T_H_ = 220 °C.
[85]	180–220	25	0.20	35–45	XY	45°/−45°	d_f_ = 1.75 mm; d_n_ = 0.4 mm; 20% infill	Tensile—ASTM D638; Bending—ASTM D790; Compression—ASTM D3410	Higher UTS for T_H_ = 220 °C; Higher compressive strength for T_H_ = 190–220 °C; Higher bending strength for T_H_ = 190–210°C.
[90]	190–210	55	0.35	40	Horizontal	0°; 45°; 90°	d_f_ = 2.85 mm; d_n_ = 0.4 mm; W_L_ = 2	Tensile—ASTM D638–10-I; s_t_ = 5 mm/min	Higher UTS and E for T_H_ = 210 °C (for all raster). Highest UTS (56.2 MPa) for specimens with T_H_ = 210 °C and θ = 0°.
[91]	180–210	60	0.20	50	XY	45/−45°	d_n_ = 0.4 mm; 100% infill; 70–160% flow	Tensile—ISO 527–2	The variation of tensile load vs. temperature is influenced by the flow rate.
[92]	210	40–80	0.20	-	Horizontal	45/−45°	d_f_ = 1.75 mm; d_n_ = 0.4 mm	Tensile—ASTM D638 Type IV specimens	Higher strength for specimens printed inside of a heated chamber.
[93]	195–255	55	0.30	45	XY	0°	d_f_ = 1.75 mm; d_n_ = 0.5 mm; 100% infill; annealing	Tensile—ISO 527; Bending—EN ISO 178:2011	Higher UTS and UFS for T_H_ = 235–255°C.
[94]	180–230	70–110	0.30	40	YZ	0°/90°	d_f_ = 1.75 mm; d_n_ = 0.4 mm; 99% infill	Tensile—ASTM D368 Type V specimens	Highest UTS (76.5 MPa) for T_H_ = 200 °C and T_B_ = 70 °C. Lowest UTS (69 MPa) for T_H_ = 180 °C and T_B_ = 110 °C.
[95]	210–230	70	0.20	40	XY	45°/−45°	d_f_ = 1.75 mm; 100% infill	Tensile—ASTM D368 Type IV specimens; s_t_ = 1 mm/min	Highest UTS (53 MPa) and E (2.5 GPa) for T_H_ = 220°C. Lowest UTS (47 MPa) and E (2.2 GPa) for T_H_ = 230 °C.

**Table 5:** The influence of the build orientation and the printing orientation angle on the mechanical properties of FFF-printed PLA.

Ref.	FFF Process Parameters							Mechanical Test Settings	Results and Conclusions
	B.O. (-)	t (mm)	s_p_ (mm/s)	T_H_ (°C)	T_B_ (°C)	θ (°)	Other Parameters		
B.O.—build orientation; t—layer thickness (layer height); s_p_—printing speed; T_H_—printing head (nozzle) temperature; T_B_—build plate temperature; θ—raster angle; d_f_—filament diameter; d_n_—nozzle diameter; W_L_—number of wall lines.									
[26]	YX; YZ; ZY	0.06–0.24	20–80	210	-	0°	d_f_ = 1.75 mm; d_n_ = 0.4 mm; 100% infill	Tensile—ASTM D638; Bending—ASTM D790	High variation of UTS and UFS. Highest values for YX and YZ specimens.
[27]	XY; XZ; ZX	0.20	-	-	-	45°/−45°	50–100% infill	Tensile—ISO 527–2; s_t_ = 10 mm/min	Highest UTS (56.5 MPa) for flat XY specimens at 100% infill. 13% and 37% decrease in UTS for XZ and ZX specimens.
[28]	XY; ZX	0.06–0.50	30–200	175–230	-	-	d_f_ = 1.75 mm; d_n_ = 0.5 mm; 100% infill; variable flow	Tensile—ASTM D638 Type I vs. Type IV	UTS for ZX specimens is 47.9% lower compared to UTS for XY specimens.
[54]	X; Y; Z	0.30	90	185	-	-	d_f_ = 1.75 mm; 100% infill	Tensile—ASTM D638 IV; s_t_ = 5 mm/min	Low variation of UTS with build orientation.
[60]	Horizontal; vertical	0.05–0.40	60	200	-	-	d_f_ = 1.75 mm; 60% infill	Tensile	UTS for vertical specimens 50% lower than UTS for horizontal specimens.
[72]	XY; XZ; ZX	0.10	30	200	50	45°/−45°	d_f_ = 1.75 mm; d_n_ = 0.4 mm; 20% infill	Tensile—ASTM D638; s_t_ = 5 mm/min	Higher UTS (38.47 MPa) for XY specimens compared to XZ (30.10 MPa) and ZX (27.63 MPa) specimens.
[83]	XY; XZ	0.08–0.28	20–60	210–220	-	0°/90°; 30°/−60°; 45°/−45°	d_n_ = 0.3–0.5 mm; 80–100 % infill; W_L_ = 2–4	Tensile—ASTM D638-I; s_t_ = 5 mm/min	Higher UTS for XZ specimens.
[97]	XY; XZ; ZX	0.40	3	220	-	-	d_n_ = 0.4 mm; 100 % infill	Tensile—ASTM D638	Highest values of E and UTS for XZ specimens.
[98]	XY; XZ; ZX	0.20	60	210	45	45°/−45°	d_f_ = 1.75 mm; d_n_ = 0.4 mm; W_L_ = 2	Tensile—ASTM D638 Type I specimens	Highest values of UTS (57.58 MPa) and E (2571 MPa) for XY specimens. Low value of UTS (23.75 MPa) for ZX specimens.
[99]	XY; XZ; ZX	0.18	80	-	-	-	d_f_ = 1.75 mm; 20–100% infill	Tensile—ASTM D638; s_t_ = 5 mm/min	Yield stress for 100% infill: XY specimens—41.66 MPa, XZ specimens—48.53 MPa, ZX specimens—24.20 MPa. Similar variation for lower infill density.
[100]	XY; XZ; ZX	0.20	50	215	60	0°; 45°; 90°	d_f_ = 1.75 mm; d_n_ = 0.4 mm; 100% infill; W_L_ = 2	Tensile—ASTM D638; s_t_ = 5 mm/min	Higher UTS (34.45–35.47 MPa) for XZ specimens. Low UTS for XY and ZX specimens. The variations are influenced by the raster.
[57]	α_XY_ = 0°- 60°	0.10–0.30	50	210	60	-	d_f_ = 1.75 mm; d_n_ = 0.4 mm; 20–80% infill; W_L_ = 2	Bending—ASTM D790; Tensile—ASTM D790; s_t_ = 1 mm/min	Low variation of the flexural strength and the tensile strength with α_XY_.
[58]	α_XY_ = 0°–45°	0.125–0.25	-	-	-	-	50–90% infill	Tensile—ISO 527–1,2	Low variation of UTS vs. the α_XY_ angle.
[59]	α_XY_ = 0°–90°	0.10–0.35	40–80	220	25	-	d_f_ = 1.75 mm; 100% infill	Tensile—ASTM D638, Type V specimens	Higher E and UTS for α_XY_ = 0° and α_XY_ = 45°.
[64]	γ_XY_ = 0°–90°	0.10–0.30	-	210	80	30°; 45°; 60°	d_f_ = 1.75 mm; 50% infill	Tensile—ASTM D638	Highest UTS for γ_XY_ = 0° and γ_XY_ = 45° specimens.
[74]	β_YZ_ = 0°–90°	0.05–0.20	60	195–230	60	-	d_f_ = 1.75 mm; d_n_ = 0.4 mm	Tensile—ISO 527–2; s_t_ = 2 mm/min	High decrease in UTS with the increase in β_YZ_.
[76]	γ_XZ_ = 0°–90°	0.10–0.30	-	215	-	-	d_f_ = 1.75 mm	Tensile—ISO 527–2	High variation of UTS with the γ_XZ_ angle, from 55.86 MPa (XZ specimens, γ_XZ_ = 0°) to 26.65 MPa (ZX specimens, γ_XZ_ = 90°).
[78]	γ_XZ_ = 0°—90°	0.10–0.60	-	-	-	-	d_f_ = 1.75 mm; d_n_ = 0.4 mm	Tensile—ISO 527–2; s_t_ = 0.1 mm/min	High variation of UTS with the γ_XZ_ angle, from 51.33 MPa (XZ specimens, γ_XZ_ = 0°) to 34.56 MPa (ZX specimens, γ_XZ_ = 90°).
[79]	γ_XZ_ = 0°–90°	0.10–0.30	-	220	60	-	d_f_ = 1.75 mm	Tensile—ISO 527–2	High variation of UTS with the γ_XZ_ angle, from 49.66 MPa (XZ specimens, γ_XZ_ = 0°) to 23.40 MPa (ZX specimens, γ_XZ_ = 90°).
[101]	α_XY_ = 0°–90°; β_YZ_ = 0°–90°; γ_XZ_ = 0°–90°	0.10	-	-	-	-	d_n_ = 0.4 mm; 99% infill	Tensile—ISO 527–2	Highest UTS (55.68 MPa) for XZ (γ_XZ_ = 0°); Low UTS (12.68–15.5 MPa) for YX, YZ, β_YZ_ = 45° and α_XY_ = 45° specimens.
[102]	γ_XY_ = 0°–90°; γ_XZ_ = 0°–90°	0.2	50	225	60	-	d_f_ = 2.75 mm; d_n_ = 0.6 mm	Tensile—ISO 527; Bending—ISO 178; Compression—ISO 604	Highest UTS (49.8 MPa) for XZ (γ_XZ_ = 0°). Lowest UTS (21.5 MPa) for ZY and ZX. UTS decreases with the increase in γ_XY_ and γ_XZ_. Low variation of the compressive strength.
[103]	γ_XY_ = 0°–90°; γ_XZ_ = 0°–90°	0.15	60	220	60	-	d_f_ = 1.75 mm; d_n_ = 0.4 mm; 25–100% infill	Tensile—ASTM D638; Shear—ASTM D5379	High decrease in UTS with the increase in γ_XZ_. UTS = 55 MPa for XZ (γ_XZ_ = 0°). Highest shear strength (36 MPa) for γ_XY_ = 45°.
[104]	β_YX_ = 0°–90°; β_XY_ = 0°–90°; β_YZ_ = 0°–90°	0.20	35	205	60	0°/90°; 30°/−60°; 45°/−45°; 60°/−30°; 90°/0°	d_f_ = 1.75 mm; d_n_ = 0.4 mm; 10% infill	Tensile—ASTM D638	Low influence of β_XY_. High influence of β_YX_ and β_YZ_. Highest UTS (27.6 MPa–30.9 MPa) for β_XY_ = 0°–90°, β_YX_ = 0° and β_YZ_ = 0° specimens.

**Table 6:** The influence of the raster angle on the mechanical properties of FFF-printed PLA.

Ref.	FFF Process Parameters							Mechanical Test Settings	Results and Conclusions
	θ (°)	t (mm)	s_p_ (mm/s)	T_H_ (°C)	T_B_ (°C)	B.O. (-)	Other Parameters		
θ—raster angle; t—layer thickness (layer height); s_p_—printing speed; T_H_—printing head (nozzle) temperature; T_B_—build plate temperature; B.O.—build orientation; d_f_—filament diameter; d_n_—nozzle diameter; W_L_—number of wall lines.									
[24]	40°; 60°; 80°	0.10–0.30	30	195	110	Horizontal	d_f_ = 1.75 mm; d_n_ = 0.3 mm; 20–80% infill	Tensile—ASTM D638	The variation of UTS vs. θ is influenced by the layer thickness.
[27]	0°; 45°; 90°	0.20	-	-	-	XY; XZ; ZX	50–100% infill	Tensile—ISO 527-2; s_t_ = 10 mm/min	A decrease of 16.7 % of the UTS for θ = 90° compared to θ = 0° and θ = 45° specimens.
[29]	0°; 30°; 45°; 60°; 90°	0.40	20–80	215	55	Horizontal	100% infill; W_L_ = 2	Tensile—ASTM D638; s_t_ = 5 mm/min	For t = 0.40 mm all specimens fractured in the direction of the raster. Highest UTS for θ = 0° specimens; UTS decreases by approx. 40% for θ = 90° specimens.
[37]	0°; 45°; 90°	0.10	30	240	60	Horizontal	d_f_ = 2.85 mm; d_n_ = 0.4 mm; 100% infill; 1–10% moisture	Tensile—ASTM D638; s_t_ = 5 mm/min	The raster angle has a high significance on UTS. Maximum UTS (50.3 MPa) and E (1890 MPa) obtained at θ = 0° and 10% moisture content.
[61]	0°; 90°	0.20–0.30	38–52	190	40	-	d_n_ = 0.40 mm; 40% infill	Bending—ASTM D790; s_t_ = 12 mm/min	A higher flexural strength for θ = 0° specimens.
[63]	0°; 18°; 45°; 72°; 90°	0.10–0.20	60	205	60	Horizontal	100% infill; W_L_ = 2–6	Tensile—ASTM D638 modified specimens	Highest UTS (53.59 MPa) and E (3388.57 MPa) for θ = 0°; Lowest UTS (43.39 MPa) and E (2799.43 MPa) for θ = 90°.
[64]	30°; 45°; 60°	0.10–0.30	-	210	80	γ_XY_ = 0°–90°	d_f_ = 1.75 mm; 50% infill	Tensile—ASTM D638	UTS decreases with the increase in θ.
[69,70,71]	0°; 45°; 90°	0.10–0.30	50	210	70	-	d_f_ = 1.75 mm; d_n_ = 0.4 mm; 100% infill; W_L_ = 1	Tensile—ASTM D638; Bending—ASTM D790; Impact—ASTM D256	High influence of the raster angle on the mechanical properties. Highest UTS, UFS and Izod impact strength for θ = 0° specimens.
[90]	0°; 45°; 90°	0.35	40	190–210	55	Horizontal	d_f_ = 2.85 mm; d_n_ = 0.4 mm; W_L_ = 2	Tensile—ASTM D638-10-I; s_t_ = 5 mm/min	Highest UTS and E for θ = 0° specimens. Lowest UTS and E for θ = 90° specimens.
[107]	0°; 30°; 45°; 60°; 90°	0.20	30	200	60	Horizontal	100% infill	Tensile—ISO 527-2, Type 1B specimens	Breaking surface aligned with the raster. Highest UTS and E for θ = 0°; UTS decreases by approx. 70% for θ = 90°.
[108]	0°; 30°; 45°; 60°; 90°	-	70	200	60	XY	d_f_ = 2.85 mm; 100% infill	Tensile—ASTM D638	High influence of the raster angle on UTS. Highest UTS for θ = 45°.
[27]	0°/45°; 45°/−45°; 0°/90°	0.20	-	-	-	XY; XZ; ZX	50–100% infill	Tensile—ISO 527-2; s_t_ = 10 mm/min	Highest UTS (58.4 MPa) for θ = 0°/45°.
[32]	45°/−45°; 0°/90°; 0°/−30°/30°/ −60°/60°/90°	0.10–0.30	60	215	60	Horizontal	d_f_ = 1.75 mm; 100% infill; ageing; heat treatment	Tensile—ASTM D638	Higher UTS for θ = −45°/45°. The variation of UTS vs. raster angle is influenced by heat treatment and ageing.
[33]	0°/90°; 15°/75°; 30/60°; 45/45°	-	50	190–230	45–105	-	d_f_ = 2.85 mm; 100% infill	Tensile—ASTM D638; s_t_ = 5 mm/min Bending—ASTM D790; Impact—ASTM D256	Highest values of tensile strength, flexural strength and Izod impact strength obtained for θ = 45/45°.
[51]	0°; 90°; 45°/−45°	0.06–0.35	60	190–220	60	XY	d_f_ = 1.75 mm; d_n_ = 0.4 mm; 100% infill; W_L_ = 2	Tensile—ASTM D638-I specimens; s_t_ = 5 mm/min	Highest UTS for θ = 45/−45°. Low values of UTS for specimens with θ = 90° and t = 0.06 mm.
[83]	0°/90°; 30°/−60°; 45°/−45°	0.08–0.28	20–60	210–220	-	XY; XZ	d_f_ = 1.75 mm; d_n_ = 0.3–0.5 mm; 80–100% infill; W_L_ = 2–4	Tensile test, ASTM D638-I; s_t_ = 5 mm/min	Higher UTS for specimens with θ = 30°/−60° and θ = 45°/−45°.
[86]	45°/−45°; 0°/90°	0.10	35–65	200	60	XY	d_f_ = 2.85 mm; 100% infill	Tensile—ASTM D638	Higher UTS for θ = 45°/−45°.
[104]	0°/90°; 30°/−60°; 45°/−45°; 60°/−30°; 90°/0°	0.20	35	205	60	β_YX_ = 0°–90°; β_XY_ = 0°–90°; β_YZ_ = 0°–90°	d_f_ = 1.75 mm; d_n_ = 0.4 mm; 10% infill	Tensile—ASTM D638	Low influence (2 MPa) of the raster angle on UTS, at 10% infill.
[107]	0°/90°; 30°/−60°; 45°/−45°	0.20	30	200	60	Horizontal	100% infill	Tensile—ISO 527-2, Type 1B specimens	Low influence of the alternating raster angle on the elastic modulus and the ultimate tensile strength.
[109]	0°/90°; 15°/-75°; 30°/−60°; 45°/−45°	0.20	55	210	60	Horizontal	-	Tensile—ASTM D638; s_t_ = 0.5 mm/min; fracture test	Highest E (1942 MPa) and yield stress (27.1 MPa) for θ = 0/90°; Highest fracture load (865.1 N) in fracture test of specimens with θ = 45/−45°.
[110]	0°; 90°; 45/−45°	0.30	50	190	55	XY	d_n_ = 0.4 mm	Tensile—ASTM D638; s_t_ = 5 mm/min	Highest UTS for θ = 0°; Low influence of the raster angle on the elastic modulus for PLA.
[111]	0°/90°; 45°/−45°	0.2	120	200	50	Horizontal	d_f_ = 1.75 mm; d_n_ = 0.4 mm; 3090 % infill; W_L_ = 2	Tensile—ISO 527	A higher strength of specimens for θ = 45°/−45°. Low influence of raster angle on elastic modulus.
[112]	0°; 90°; 45°/0°/90°/ 135°	0.14	40	215	60	XY	d_f_ = 1.75 mm; d_n_ = 0.4 mm; 100% infill	Tensile—ASTM D638	Highest UTS (57.7 MPa) for θ = 0°; Lowest UTS (30.8 MPa) for θ = 90°.
[113]	45°/−45°; 0°/90°	0.15	40	210	50	-	d_f_ = 1.75 mm; d_n_ = 0.5 mm; 100% infill	Bending—ASTM D790; Compression—ASTM S695; Impact test—ASTM D256	Higher UFS (+14.31%) and impact strength (+41.20%) for θ = 45°/−45°. Low influence of raster angle on the compressive strength.
[114]	45°/−45°; 0°/90°	0.25	50	210	60	XY	d_f_ = 1.75 mm; d_n_ = 0.4 mm; 100% infill	Tensile—ASTM D638; Bending—ASTM D790; Impact—ASTM D256	Higher UTS and Izod impact strength for θ = 45°/−45°; Higher UFS for θ = 0°/90°.

## Data Availability

Not applicable.
